# Apneic Oxygenation: A Summarized Review and Stepwise Approach

**DOI:** 10.7759/cureus.50916

**Published:** 2023-12-21

**Authors:** Mohamed Fayed, Wissam Maroun, Nimesh Patel, Dragos Galusca

**Affiliations:** 1 Cardiothoracic Anesthesia, Montefiore Medical Center, Bronx, USA; 2 Anesthesiology, Pain Management and Perioperative Medicine, Henry Ford Health System, Detroit, USA; 3 Anesthesiology, Pain Management and Perioperative Medicine, Massachusetts General Hospital, Harvard Medical School, Boston, USA; 4 Anesthesia and Critical Care, Henry Ford Health System, Detroit, USA

**Keywords:** oxygen therapy, high flow nasal cannula (hfnc), can't intubate can't ventilate, laryngeal pathology, stepwise approach, emergency endotracheal intubation, desaturation, difficult airway management, airway intubation, apneic oxygenation

## Abstract

Apneic oxygenation is a technique used during airway management procedures to maintain oxygenation and prevent desaturation during a lack of ventilation. Despite its importance, there is a lack of comprehensive information on how to achieve effective apneic oxygenation, leading to misunderstandings and suboptimal utilization of this technique. Apneic oxygenation involves several key steps. Firstly, patient selection is crucial, considering factors such as anticipated difficulty with airway management, reduced functional residual capacity, increased oxygen consumption, and medical conditions associated with impaired oxygenation. Secondly, adequate preoxygenation is essential to optimize oxygen reserves before the onset of apnea, utilizing methods like non-rebreather oxygen masks or specific breathing techniques. Thirdly, maintaining airway patency through techniques such as jaw thrust or nasopharyngeal airway placement allows for unobstructed airflow during the apneic period. Lastly, the selection of the appropriate oxygen delivery method, such as high-flow nasal oxygen or nasal cannula, depends on the patient's existing respiratory support. Despite the growing body of literature on apneic oxygenation, current review articles often lack a stepwise approach for its proper execution. This knowledge gap contributes to the misunderstanding and underutilization of this important tool during intubation and airway management. In conclusion, apneic oxygenation is a valuable technique for maintaining oxygenation during periods of apnea. However, the lack of comprehensive information and stepwise guidance in the current literature hinders its optimal utilization. Clear guidelines and educational resources should be developed to address this knowledge gap and ensure the safe and effective implementation of apneic oxygenation. By following a stepwise approach that includes patient selection, adequate preoxygenation, airway patency, and appropriate oxygen delivery, healthcare providers can enhance patient outcomes and minimize the risk of desaturation during airway management procedures.

## Introduction and background

History of apneic oxygenation

The history of apneic oxygenation dates back to a study conducted in 1959. In this study, a group of eight healthy patients was scheduled for minor surgeries. The researchers aimed to examine the effectiveness of apneic oxygenation during periods of apnea [1]. Before the induction of anesthesia, the patients were oxygenated with 100% oxygen using a circle anesthesia system for five minutes. Following this, they received hypnotic doses of thiopental and succinylcholine to induce unconsciousness and ensure that the patients were not breathing spontaneously. To denitrogenate the patients' lungs and prepare for apneic oxygenation, they were exposed to 100% oxygen for at least 30 minutes before the induction of anesthesia. Additional doses of thiopental and succinylcholine or d-tubocurarine were administered to maintain unconsciousness and prevent any respiratory effort. The study's results indicated that all eight patients could maintain apnea for a duration ranging from 18 minutes to 55 minutes. Throughout this period, their oxygen saturation levels remained above 98%, demonstrating successful oxygenation without a drop in oxygen levels.

Physiological mechanism of apneic oxygenation

Before the onset of apnea, preoxygenation is typically performed to denitrogenate the patient's lungs. This involves breathing in oxygen for a sufficient duration to replace nitrogen in the alveoli with oxygen. By removing nitrogen, the oxygen concentration in the alveoli is increased, setting the stage for apneic oxygenation. Apneic oxygenation relies on the principle of "aventilatory mass flow" or "apneic diffusion of oxygenation," which involves the movement of additional oxygen into the alveoli due to a subatmospheric pressure generated by the diffusion of oxygen from the alveoli into the bloodstream [2].

**Figure 1:**
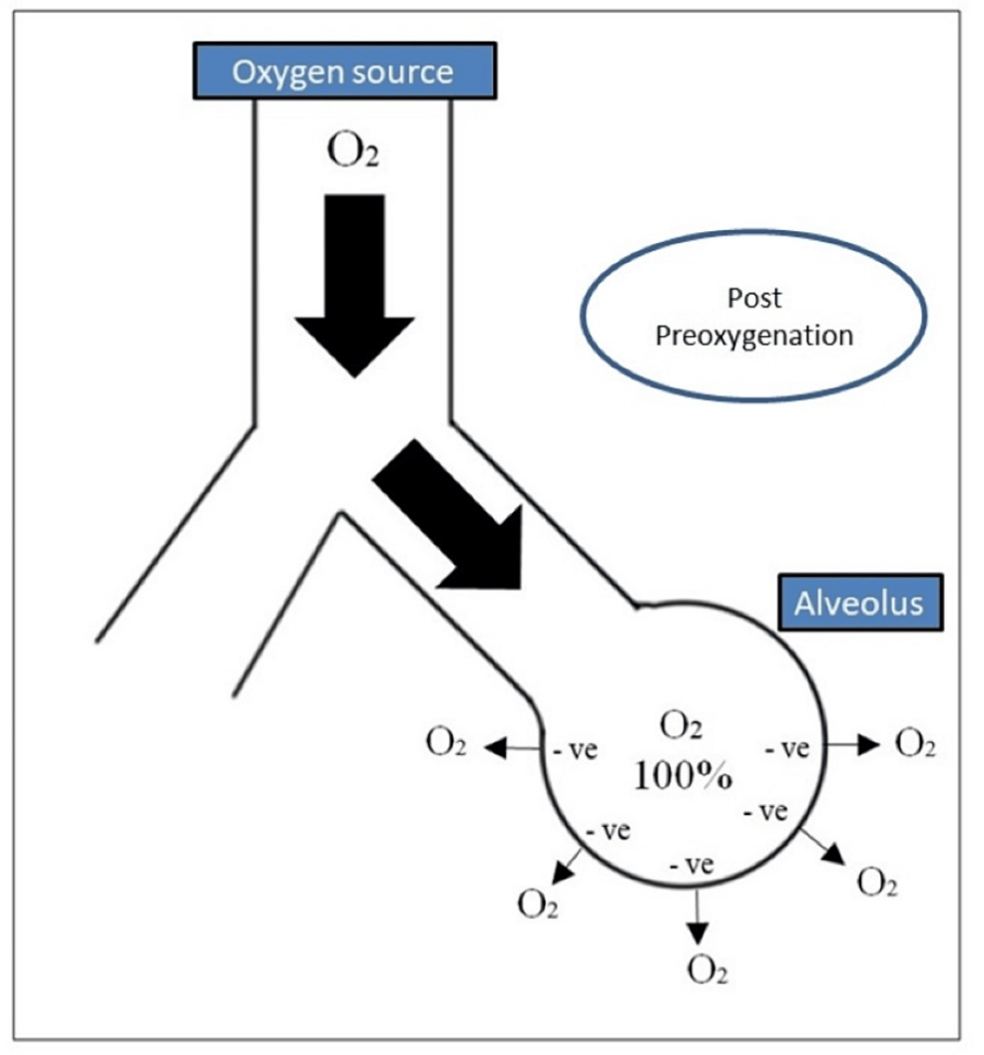
Apneic Oxygenation Post-preoxygenation O_2_: oxygen, -ve: negative pressure

During apnea, oxygen continues to diffuse from the alveoli into the blood. This extraction process causes the pressure in the alveoli to become subatmospheric, creating a pressure gradient. This pressure gradient allows for the movement of additional oxygen from the administered oxygen source (such as a mask or nasal cannula) into the alveoli. The subatmospheric alveolar pressure created during apnea facilitates the flow of oxygen into the alveoli, even in the absence of active ventilation (Figure 1) [3].

**Figure 2:**
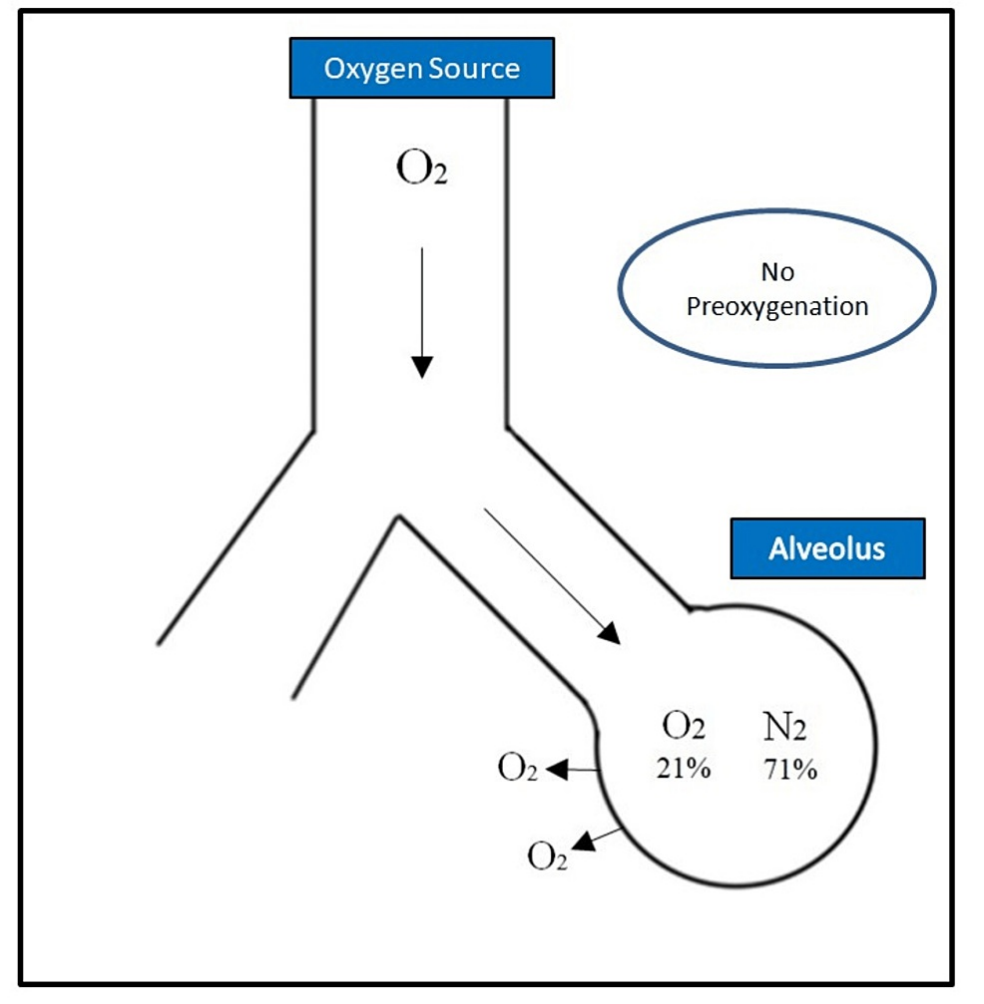
Apneic Oxygenation Without Preoxygenation O_2_: oxygen, N_2_: nitrogen

This helps to maintain oxygenation and prevent the onset of hypoxemia, which can occur rapidly if the preoxygenation is inadequate. If preoxygenation is not sufficient, the presence of nitrogen in the lungs, along with accumulating carbon dioxide, reduces the pressure gradient available for oxygen transfer to the alveoli. This diminishes the effectiveness of apneic oxygenation and hastens the onset of hypoxemia (Figure 2) [4].

## Review

The technique of apneic oxygenation

The technique of apneic oxygenation involves specific steps to ensure effective oxygenation during periods of apnea:

1. Preoxygenation

Before the induction of anesthesia, ensure that the patient is pre-oxygenated. This can be done using a non-rebreather oxygen mask at a flow rate of 15 liters per minute or by connecting the patient to an anesthesia machine ventilatory circuit. Preoxygenation helps denitrogenate the lungs and maximize the oxygen reservoir.

2. Induction of Anesthesia

Once preoxygenation is complete, administer the chosen induction agent to induce anesthesia and initiate apnea.

3. Maintain the Nasal Cannula Flow Rate

During the apneic period, it is important to maintain the flow rate of oxygen via a nasal cannula. This ensures a continuous supply of oxygen to the upper airway.

4. Maintain a Patent Airway

It is crucial to keep the patient's airway open and patent until the time of intubation. This can be achieved using techniques such as jaw thrust, and/or nasopharyngeal airway. These maneuvers help prevent airway obstruction and ensure the passive movement of oxygen from the upper airway to the trachea.

5. Continue Oxygenation During Intubation

Even during the process of intubation, it is important to continue delivering oxygen via the nasal cannula (NC) or high-flow nasal oxygen (HFNO). This ensures that oxygenation is maintained throughout the procedure.

Pitfalls to avoid during apneic oxygenation

1. Failing to Maintain Airway Patency

If the airway is not kept open, the passive movement of oxygen from the nasal cannula to the trachea may be compromised, affecting the effectiveness of apneic oxygenation.

2. Inadequate Preoxygenation

Insufficient preoxygenation can diminish the efficacy of the passive flow of oxygen from the upper airway to the alveoli, as described in the physiological mechanism earlier.

3. Relying on an Ambu Bag

**Figure 3:**
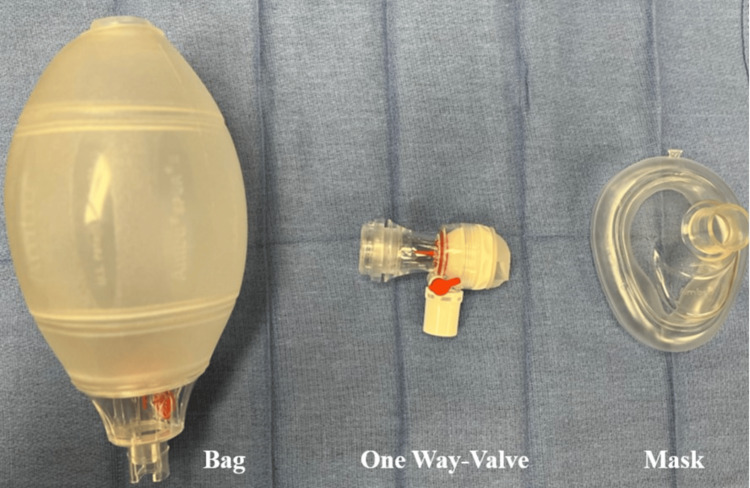
Different Components of Ambu Bag

**Figure 4:**
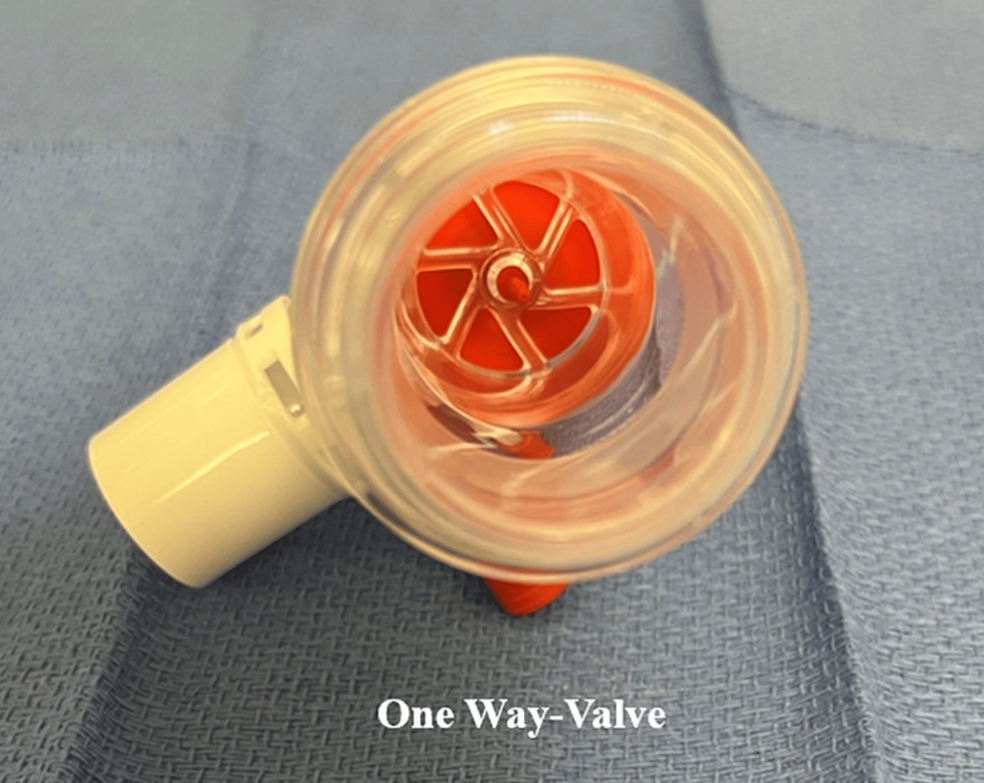
A Closer Look at the One-Way Valve

An Ambu bag or manual resuscitator with a one-way valve should not be used as the sole method of apneic oxygenation. The one-way valve prevents the passive flow of oxygen to the mask, and attempting to provide active breaths during intubation poses risks such as gastric distension and aspiration (Figures 3-4).

Evidence of apneic oxygenation

We summarized six important studies looking at apneic oxygenation:

1. Apneic Oxygenation Using NC in Obese Patients

The study conducted by Ramachandran et al. at the University of Michigan in 2010 provided evidence supporting the effectiveness of apneic oxygenation [5]. This was a non-blinded randomized controlled trial (RCT) involving obese patients scheduled for surgery. The mean body mass index (BMI) of the participants was 31 kg/m^2^, and the total number of patients included in the study was 30. The patients were randomly assigned to two groups. The apneic oxygenation group received NC attached to five liters per minute of 100% fraction of inspired oxygen (FiO_2_), while the control group received NC with room air. The primary outcomes measured were the duration and number of patients with oxygen saturation (SpO_2_) equal to or above 95% at a maximum of six minutes. The minimum SpO_2_ was also compared between the two groups. The apneic oxygenation group demonstrated significant improvements in oxygenation parameters compared to the control group. The apneic oxygenation group had a significant prolongation of SpO_2_ ≥95% time (5.29 minutes versus 3.49 minutes) and a significant increase in the number of patients with SpO_2_ ≥95% at the six-minute mark (eight patients versus one patient). Additionally, the apneic oxygenation group had a significantly higher minimum SpO_2 _(94.3% versus 87.7%) compared to the control group.

2. Apneic Oxygenation Using HFNO for Hypopharyngeal or Laryngotracheal Surgery

The study conducted in London in 2014, titled *Transnasal Humidified Rapid-Insufflation Ventilatory Exchange (THRIVE)*, involved 25 patients who underwent general anesthesia for hypopharyngeal or laryngotracheal surgery. Out of the 25 patients, 12 were obese, and nine patients presented with stridor [6]. During the procedure, the patients received the THRIVE technique. This technique involved the continuous delivery of transnasal high-flow humidified oxygen. Initially, the oxygen was administered for pre-oxygenation purposes, and it continued to be provided as post-oxygenation during the intravenous induction of anesthesia and neuromuscular blockade until a definitive airway was established. The airway was maintained open using a jaw thrust maneuver. The median apnea time observed in the study was 14 minutes, with a range of 5 to 65 minutes. Importantly, none of the patients experienced arterial desaturation below 90% throughout the procedure. This indicates that the use of THRIVE with transnasal high-flow humidified oxygen effectively maintained adequate oxygenation during the apnea period, ensuring patient safety and preventing hypoxia.

3. Apneic Oxygenation Using NC in the Medical Intensive Care Unit

This trial, conducted in 2015, was a single-center, Vanderbilt University USA [7]. It is an RCT in a medical intensive care unit setting. The study aimed to compare the effectiveness of apneic oxygenation versus usual care during intubation in critically ill patients. The trial included 150 patients who were intubated by a pulmonary and critical care medicine fellow at Vanderbilt, with an additional 46 patients excluded for various reasons. The intervention group (n=73) received apneic oxygenation, while the control group (n=73) received usual care. Furthermore, patients were randomized to either video laryngoscopy or direct laryngoscopy for intubation. The outcomes measured in the study included various parameters related to oxygenation and patient outcomes.

The results indicated that the median lowest arterial oxygen saturation was 92% with apneic oxygenation versus 90% with usual care (95% CI, -1.6 to 7.4%; P = 0.16). There was no difference between apneic oxygenation and usual care in the incidence of SpO_2_ less than 90% (44.7 vs. 47.2%; P = 0.87), SpO_2_ less than 80% (15.8 vs. 25.0%; P = 0.22), or decrease in SpO_2_ greater than 3% (53.9 vs. 55.6%; P = 0.87).

This trial had several limitations that should be considered when interpreting the results. Firstly, the study excluded patients who required urgent intubation. This exclusion criterion may limit the generalizability of the findings to a broader population, particularly those who are critically ill or have difficult airways. Secondly, the trial reported that 75% of patients were classified as having easy intubation. Benefits from apneic oxygenation appear in difficult intubation where a longer time is needed to secure the airway. Thirdly, the study mentioned that 73% of patients received bag-mask ventilation until the time of intubation. This practice does not align with the concept of apneic oxygenation, which involves the administration of oxygen without positive pressure ventilation during intubation. The inclusion of patients who received bag-mask ventilation may confound the effects of true apneic oxygenation. Finally, the study did not collect data on the patency of the airway during intubation. This information could have provided insights into the effectiveness of apneic oxygenation in maintaining airway oxygenation and patency during the intubation process.

4. Apneic Oxygenation Using HFNO in Morbidly Obese Patients

In a study conducted by Wong et al., the researchers aimed to simulate the study previously discussed by Ramachandran but specifically recruited morbidly obese patients with a BMI greater than or equal to 40 kg/m^2^. The study included a population of 40 patients meeting these criteria [8]. The intervention in the study was HFNO at a flow rate of 60 L/min. The researchers also simulated a difficult airway scenario to assess the impact of HFNO on safe apnea time. The primary outcome of the study was the safe apnea time, defined as the duration until either the SpO_2_ dropped below 95% or a maximum of six minutes of apnea.

The results of the study showed the following findings when comparing the HFNO group with the simulated difficult airway:

Safe apnea time: The safe apnea time was significantly longer in the HFNO group, with a mean duration of 4.3 minutes compared to three minutes in the control group (P = 0.001).

Minimum peri-intubation SpO_2_: The HFNO group exhibited a higher minimum peri-intubation SpO_2_ compared to the control group. The minimum SpO_2_ was 91% in the HFNO group and 88% in the control group (P = 0.026).

5. Meta-Analysis on Apneic Oxygenation in ICU Setting

The meta-analysis conducted by Binks et al. in 2017 examined the use of apneic oxygenation in the intensive care unit setting. The analysis included a total of six studies with a combined sample size of 518 patients [9]. The meta-analysis showed a reduction in the occurrence of critical desaturation events in patients who received apneic oxygenation compared to those who did not. The relative risk of critical desaturation was 0.69, indicating a 31% reduction in the risk of experiencing critical desaturation events. However, the 95% confidence interval (CI) ranged from 0.48 to 1. The analysis also revealed a statistically significant increase in the lowest SpO_2_ value in patients who underwent apneic oxygenation. The lowest SpO_2_ value increased by an average of 2.83%, with a 95% CI ranging from 2.28% to 3.38%. This indicates that apneic oxygenation led to a significant improvement in the minimum SpO_2_ levels achieved during the apneic period.

6. Meta-Analysis on Apneic Oxygenation During Emergency Intubation

The study by Silva et al. in 2017 focused on the use of apneic oxygenation during emergency intubation. The meta-analysis included eight studies with a total of 1,837 patients [10]. The analysis revealed that apneic oxygenation during emergency intubation led to a statistically significant increase in peri-intubation SpO_2_ levels. The weighted mean difference was 2.2%, indicating an average increase in SpO_2_ during the intubation process. The meta-analysis also showed that the use of apneic oxygenation resulted in a decreased risk of hypoxemia during emergency intubation. The odds ratio for experiencing hypoxemia was 0.66, with a 95% CI ranging from 0.52 to 0.84. The analysis also indicated that apneic oxygenation was associated with an increased rate of first-pass intubation success. The odds ratio for achieving successful intubation on the first attempt was 1.59, with a CI of 1.04 to 2.44. These findings highlight the benefits of utilizing apneic oxygenation during emergency intubation procedures. It can lead to increased peri-intubation SpO_2_, decreased rates of hypoxemia, and improved first-pass intubation success.

Recommendations

**Figure 5:**
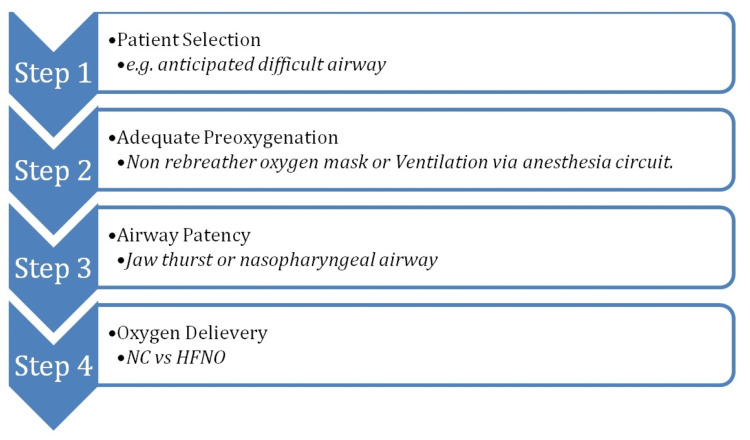
Summary of Recommendations NC: nasal cannula, HFNO: high-flow nasal oxygenation

Our recommended step-wise approach for the use of apneic oxygenation (Figure 5):

Step 1: Patient Selection

Identify patients who are likely to benefit from apneic oxygenation. This includes patients with anticipated difficulty with airway management [11], reduced functional residual capacity, increased oxygen consumption, and medical conditions associated with reduced oxygenation.

Step 2: Adequate Preoxygenation

For emergent intubations, use a non-rebreather oxygen mask and increase the oxygen flow meter to 15 L/min [12]. For elective or non-urgent cases, perform tidal volume breathing for at least three minutes [13] or deliver eight vital capacity breaths over one minute. The aim is to achieve an end-tidal oxygen concentration above 90% [14].

Step 3: Maintain Airway Patency

During the apneic period, ensure the airway remains patent. This can be achieved using techniques such as jaw thrust or nasopharyngeal airway placement.

Step 4: Select the Oxygen Delivery Method

If the patient is already on HFNO, continue using it with a flow rate of 50-60 L/min and increase the fraction of FiO_2_ to 100%. If the patient is not on HFNO, consider using a nasal cannula at a flow rate of five liters per minute or adjust the oxygen flow rate accordingly. The advantages and disadvantages are summarized in Table 1.

**Table 1:** Comparison between HFNO and NC BMV: bag-mask ventilation, ETO_2_: end-tidal oxygen concentration, HFNO: high-flow nasal oxygenation, NC: nasal cannula

	HFNO	Nasal Cannula
Advantages	Can provide pre-oxygenation	Cheap and available
	Freehand	Can provide BMV if needed
	Might be more effective in laryngeal pathologies	Sample ETO_2_
Disadvantages	Relatively expensive	Can’t provide pre-oxygenation
	Not widely available	
	Can’t provide BMV	Might not be as effective as HFNO in laryngeal pathologies
	Can’t sample ETO_2_	

Special consideration for patients with laryngeal pathologies: For patients with laryngeal pathologies, assess the risks and benefits of using HFNO. Individualize the approach based on the patient's specific condition and the potential impact on airway patency and oxygenation. It's important to note that this step-wise approach provides general guidance, and the specific management may vary based on the patient's clinical condition, institutional protocols, and the healthcare provider's expertise. Always follow the established guidelines and consult with a healthcare professional for personalized recommendations.

In the COVID-19 era, the timing of intubation and bag-mask ventilation plays an important role [15]. Bag-mask ventilation should be avoided in COVID-19 patients when possible. Prior studies have found that manual ventilation before intubation was associated with an increased risk of SARS transmission and poses a similar risk for COVID-19 transmission [16]. Hence, apneic oxygenation will play an important role in this aspect.

## Conclusions

Apneic oxygenation is a valuable technique that can help maintain oxygenation during periods of apnea, reducing the risk of desaturation and improving patient safety during airway management procedures. It involves patient selection, adequate preoxygenation, maintaining airway patency, and selecting the appropriate oxygen delivery method. However, there is a lack of comprehensive stepwise approaches in current review articles, leading to a misunderstanding of this important tool during intubation. By implementing this technique effectively, healthcare professionals can enhance patient outcomes and reduce the complications associated with airway management.
